# Building a predictive model of low birth weight in low- and middle-income countries: a prospective cohort study

**DOI:** 10.1186/s12884-023-05866-1

**Published:** 2023-08-22

**Authors:** Jackie K. Patterson, Vanessa R. Thorsten, Barry Eggleston, Tracy Nolen, Adrien Lokangaka, Antoinette Tshefu, Shivaprasad S. Goudar, Richard J. Derman, Elwyn Chomba, Waldemar A. Carlo, Manolo Mazariegos, Nancy F. Krebs, Sarah Saleem, Robert L. Goldenberg, Archana Patel, Patricia L. Hibberd, Fabian Esamai, Edward A. Liechty, Rashidul Haque, Bill Petri, Marion Koso-Thomas, Elizabeth M. McClure, Carl L. Bose, Melissa Bauserman

**Affiliations:** 1 Department of Pediatrics, University of North Carolina at Chapel Hill School of Medicine, 101 Manning Dr, Chapel Hill, NC 27514 USA; 2 RTI International, Research Triangle Park, Durham, NC USA; 3 Kinshasa School of Public Health, University of Kinshasa, Kinshasa, Democratic Republic of the Congo; 4 Jawaharlal Nehru Medical College, KLE University, Belagavi, India; 5 Department of Obstetrics and Gynecology, Thomas Jefferson University, Philadelphia, PA USA; 6 University Teaching Hospital, Lusaka, Zambia; 7 University of Alabama at Birmingham, Birmingham, AL USA; 8 Institute of Nutrition of Central America and Panama, Guatemala City, Guatemala; 9 School of Medicine, University of Colorado, Aurora, CO USA; 10 Department of Community Health Sciences, Aga Khan University, Karachi, Pakistan; 11 Department of Obstetrics and Gynecology, Columbia University, New York, NY USA; 12 Lata Medical Research Foundation, Nagpur & Datta Meghe Institute of Medical Sciences, Sawangi, India; 13 School of Public Health, Boston University, Boston, MA USA; 14 Department of Child Health and Paediatrics, School of Medicine, Moi University, Eldoret, Kenya; 15 School of Medicine, Indiana University, Indianapolis, IN USA; 16 International Centre for Diarrhoeal Disease Research, Dhaka, Bangladesh; 17 Division of Infectious Diseases, University of Virginia, Charlottesville, VA USA; 18 Eunice Kennedy Shriver National Institute of Child Health and Human Development, National Institutes of Health, Bethesda, MD USA

**Keywords:** Low birth weight, Preterm, Small for gestational age, Low-income country

## Abstract

**Background:**

Low birth weight (LBW, < 2500 g) infants are at significant risk for death and disability. Improving outcomes for LBW infants requires access to advanced neonatal care, which is a limited resource in low- and middle-income countries (LMICs). Predictive modeling might be useful in LMICs to identify mothers at high-risk of delivering a LBW infant to facilitate referral to centers capable of treating these infants.

**Methods:**

We developed predictive models for LBW using the NICHD Global Network for Women’s and Children’s Health Research Maternal and Newborn Health Registry. This registry enrolled pregnant women from research sites in the Democratic Republic of the Congo, Zambia, Kenya, Guatemala, India (2 sites: Belagavi, Nagpur), Pakistan, and Bangladesh between January 2017 – December 2020. We tested five predictive models: decision tree, random forest, logistic regression, K-nearest neighbor and support vector machine.

**Results:**

We report a rate of LBW of 13.8% among the eight Global Network sites from 2017–2020, with a range of 3.8% (Kenya) and approximately 20% (in each Asian site). Of the five models tested, the logistic regression model performed best with an area under the curve of 0.72, an accuracy of 61% and a recall of 72%. All of the top performing models identified clinical site, maternal weight, hypertensive disorders, severe antepartum hemorrhage and antenatal care as key variables in predicting LBW.

**Conclusions:**

Predictive modeling can identify women at high risk for delivering a LBW infant with good sensitivity using clinical variables available prior to delivery in LMICs. Such modeling is the first step in the development of a clinical decision support tool to assist providers in decision-making regarding referral of these women prior to delivery. Consistent referral of women at high-risk for delivering a LBW infant could have extensive public health consequences in LMICs by directing limited resources for advanced neonatal care to the infants at highest risk.

**Supplementary Information:**

The online version contains supplementary material available at 10.1186/s12884-023-05866-1.

## Background

More than 20 million low birth weight (LBW, < 2500 g) infants are born annually [1]. LBW infants are at increased risk for mortality and serious neurodevelopmental outcomes, making LBW a major global public health problem [2]. In addition to mortality risks, LBW infants often need advanced medical care after birth to treat problems associated with prematurity (e.g., respiratory distress syndrome, infections, feeding problems) or problems associated with being born small for gestational age (SGA; e.g., hypoglycemia, hypothermia, poor postnatal growth). Since few centers in low- and middle-income countries (LMICs) have the ability to provide advanced neonatal care, allocation of advanced care towards LBW infants is a critical part of improving health outcomes for this population.

Identification of pregnant women at risk for the delivery of LBW infants prior to birth could facilitate referral of these women to delivery centers with advanced neonatal care, thereby reducing neonatal mortality related to LBW. Machine learning, or predictive modeling, has been successful at identifying high-risk groups for certain health outcomes, [3–6] and therefore could be a useful tool to risk-stratify pregnant women in low-resource settings [7]. If a machine learning tool could reliably predict women with pregnancies at high risk of LBW, it could be produced in a user-friendly interface to help providers make decisions about referral of these women to delivery centers with advanced neonatal care. Prior studies have investigated the use of machine learning techniques for the prediction of birth weight, but the majority have used small datasets ranging from less than 100 to 50,000 women [8–27]. Predictive modeling tools based on high quality data from larger data sets are needed to more accurately predict LBW in low-resource settings.

The *Eunice Kennedy Shriver* National Institute of Child Health and Human Development Global Network for Women’s and Children’s Health Research (GN) maintains a Maternal and Newborn Health Registry (MNHR) documenting pregnancy characteristics and outcomes for over 30,000 mother/infant dyads annually in seven LMICs. This high quality and large dataset is a unique resource to investigate predictive models for LBW in low-resource settings. For this study, our goal was to determine pregnancy characteristics associated with greater probability of delivering LBW infants using the GN MNHR dataset. We also aimed to develop and compare the performance of five predictive models to identify LBW infants using the MNHR data. Understanding these predictors may assist in identifying who will need additional care at delivery, facilitating timely advanced care for LBW infants and thereby reducing long-term morbidity and mortality. We hypothesized that predictive model analysis would identify previously-known prenatal predictors associated with LBW (e.g., infection and hypertension/eclampsia) and new predictors not previously considered or fully explored in prior analyses.

## Methods

We used the GN MNHR dataset for this study, which includes data from eight GN research sites in seven LMICs (the Democratic Republic of the Congo, Zambia, Kenya, Guatemala, India [2 sites: Belagavi, Nagpur], Pakistan, and Bangladesh) [28]. The MNHR contains maternal, pregnancy and delivery characteristics collected by trained research staff using medical record abstraction and in-person interviews with pregnant women. In the MNHR, birthweights are measured on all livebirths and stillbirths, and fresh stillbirths are defined as having no signs of maceration, such as skin or soft tissue changes including skin sloughing or discoloration. The MNHR is approved by appropriate institutional review boards or research ethics committees at each participating institution. The MNHR undergoes routine quality assurance processes [29] and is registered as trial number NCT01073475 in clinicaltrials.gov.

For this study, we included singleton livebirths and fresh stillbirths in the GN MNHR who were not lost to follow-up prior to delivery and delivered at or after 20 weeks (in keeping with the MNHR definition of stillbirth occurring at or after 20 weeks) between January 2017 and December 2020 [28, 30]. We excluded maternal deaths prior to delivery, miscarriages, medical terminations of pregnancy (MTP), macerated stillbirths or stillbirth of unknown type, unknown birth outcomes, multiples, LBW status missing and births with any predictive model covariate missing.

### Outcome and variable definitions

Our primary outcome was LBW, defined as birth weight < 2500 g by measured weight when available, or estimated weight. We selected LBW as a surrogate for preterm birth given the lack of reliable gestational age dating for the total birth population. We evaluated candidate predictors from the variables that are collected in the MNHR, focusing on characteristics that do not require the use of lab tests or ultrasound which may not be available in all resource-poor settings. We selected characteristics or complications that were present prior to the time of delivery since our focus was to build a predictive model that could direct care prior to delivery. We evaluated maternal characteristics of age (< 20 years old, 20–35 years old, > 35 years old), education (no formal education, primary/secondary education, University +), parity (0, 1, 2, 3, 4 +), height, maternal weight, socioeconomic status (SES) score (< 34, 34–65, 66 + , where lower scores indicate lower household assets and SES status) [31] and previous livebirth (yes, no, no previous pregnancy lasting 20 + weeks). Of note, SES data collection in the MNHR was initiated in 2017 but site initiation varied throughout the year. We also evaluated pregnancy characteristics including the number of antenatal care visits (0, 1–3, 4 +), use of iron supplementation, use of vitamin or calcium supplementation, hypertensive disorders (systolic blood pressure ≥ 140 mmHg and diastolic blood pressure ≥ 90 mmHg on two or more occasions after 20 weeks of pregnancy, proteinura, or generalized seizures in the setting of preeclampsia), severe antepartum hemorrhage (vaginal bleeding after 22 weeks of pregnancy and before the onset of labor that is > 1,000 mL or heavy enough to soak a pad or cloth in less than five minutes), and severe infection during pregnancy (serious illness with symptoms that can include fever, chills, rapid breathing, rapid heart rate, confusion, disorientation, hypotension, and cold, clammy skin).

### Analytic methods

We completed exploratory data analysis of study outcomes, maternal characteristics, and pregnancy characteristics, looking for predictors that were highly correlated with each other, had no or little variation or were missing for many subjects. We generated descriptive statistics of frequencies for categorical variables and count, mean, and standard deviation for continuous variables.

We prepared data for the models to exclude participants missing one or more of the predictors. The binary outcome for the predictive models was LBW. The variables described above were included as predictors. We prepared data and descriptive tables using SAS 9.4 and ran predictive models using *Scikit-learn* in Python 3. We picked Belagavi as the reference because this site generally enrolled women earlier than the other sites, thus representing ‘best case scenario’ for having information available early in pregnancy. We picked parity of one as the reference since nulliparous women are at greater risk for poor outcomes and of the remaining parity groups, parity = 1 had the largest sample size.

We developed and tested five predictive models: decision tree and random forest (both tree-based models), logistic regression, K-nearest neighbors and support vector machines. The decision tree model, based on classification and regression trees (CART), splits the subjects into consecutive sub-groups based on the most important predictors at each node of the tree. The random forest model avoids overfitting that may happen with a decision tree model by using multiple trees. For both tree-based models, we used Gini impurity criterion to determine which predictors yielded the most information for each classification split. Thirdly, we employed a regularized logistic regression model, which used an L2 ridge regulation penalty to avoid overfitting. For our fourth model, we used K-nearest neighbors with weights set by distance. Finally, our fifth model type was support vector machine models where we ran linear, degree 2 polynomial and radial basis kernel functions. For all models, except the K-nearest neighbors, we improved the class imbalance in the study outcome by using balanced weights.

To develop the models, we split the data into a training dataset (75% of available data) and test dataset (25% of available data). Hyperparameters were tuned using tenfold grid-search cross validation with scoring = ’roc_auc’. In addition to hyperparameter tuning, we varied the cut point for the probability used to classify an outcome as LBW for the logistic regression model from 0.1 to 0.9. We trained the models on the training data and validated the models on the test data. To validate the models, we generated predictive accuracy measures, including calculating area under the curve (AUC) and producing receiver operator characteristic (ROC) curves. We calculated precision (positive predictive value), recall (sensitivity), and f1 scores using the classification_report() method. To further evaluate model performance, we generated calibration curves. We implemented the permutation-based importance in Scikit-Learn as permutation_importance() method. This method randomly shuffles each feature and computes the change in the model’s performance. The features which impact the performance the most are the most important ones. Additionally, we created partial dependency plots of the probability of LBW based on the predictors for the models that performed the best.

## Results

**Fig. 1:**
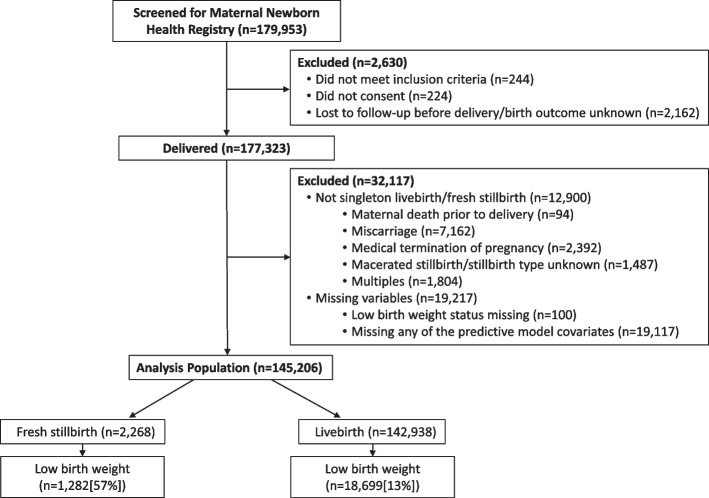
CONSORT diagram depicting reasons for exclusion and outcome for analysis population

Of the 179,953 women screened in the MNHR from January 2017 to December 2020, 145,206 (80.7%) women were eligible, consented, were not lost to follow-up prior to delivery, and delivered a singleton fresh stillbirth or livebirth with known LBW status and non-missing covariates (Fig. 1, Table 1). The most common reasons for exclusion were miscarriage and MTP in the Asian sites (total of 17.7% of Belagavi, 7.1% of Nagpur, 9.4% of Pakistani and 4% of Bangladeshi deliveries). The most common missing covariates were SES (11.2% missing overall) and maternal height (4% of subjects in Kenya) (data not shown). Other exclusions (maternal death prior to delivery, macerated stillbirth or unknown stillbirth type, unknown birth outcome, multiples, and LBW status missing) occurred in < 2% of deliveries at each site. Of the analysis subset, 2,268 were fresh stillbirths and 142,938 were livebirths; 13.8% were LBW (of which 98.9% were measured weights). The Asian sites had the highest LBW rates of 19.2% or more, and Zambia and Kenya had the lowest rates at 6.4% and 3.8%, respectively.

**Table 1:** Screening, exclusion and outcome characteristics for analysis population from 2017 – 2020

	**Overall**	**DRC**	**Zambia**	**Kenya**	**Guatemala**	**Belagavi**	**Nagpur**	**Pakistan**	**Bangladesh**
Screened, N	179,953	24,914	22,184	26,881	30,010	23,986	23,274	23,112	5,592
Ineligible, N (%)	244 (0.1)	0 (0.0)	0 (0.0)	0 (0.0)	0 (0.0)	0 (0.0)	0 (0.0)	0 (0.0)	244 (4.4)
Did not consent, N (%)	224 (0.1)	0 (0.0)	0 (0.0)	1 (0.0)	202 (0.7)	3 (0.0)	0 (0.0)	0 (0.0)	18 (0.3)
Lost to follow-up prior to delivery or missing delivery outcome, N (%)	2,162 (1.2)	258 (1.0)	66 (0.3)	1,387 (5.2)	239 (0.8)	5 (0.0)	60 (0.3)	104 (0.4)	43 (0.8)
Delivered, N (% of screened)	177,323 (98.5)	24,656 (99.0)	22,118 (99.7)	25,493 (94.8)	29,569 (99.2)	23,978 (100.0)	23,214 (99.7)	23,008 (99.6)	5,287 (99.2)
Excluded ^a^, N (%)	32,117 (18.1)	2,342 (9.5)	2,668 (12.1)	2,886 (11.3)	4,697 (15.9)	8,365 (34.9)	3,593 (15.5)	7,144 (31.1)	422 (8.0)
Did not meet singleton livebirth / fresh stillbirth criteria, N (%)	12,900 (7.3)	1,127 (4.6)	515 (2.3)	832 (3.3)	918 (3.1)	4,551 (19.0)	1,950 (8.4)	2,715 (11.8)	292 (5.5)
Maternal death prior to delivery, N (%)	94 (0.1)	29 (0.1)	9 (0.0)	13 (0.1)	8 (0.0)	7 (0.0)	9 (0.0)	18 (0.1)	1 (0.0)
Miscarriage, N (%)	7,162 (4.0)	237 (1.0)	136 (0.6)	241 (0.9)	571 (1.9)	2,829 (11.8)	1,033 (4.4)	1,914 (8.3)	201 (3.8)
MTP, N (%)	2,392 (1.3)	43 (0.2)	4 (0.0)	21 (0.1)	15 (0.1)	1,424 (5.9)	629 (2.7)	247 (1.1)	9 (0.2)
Macerated stillbirth or stillbirth type unknown, N (%)	1,487 (0.8)	396 (1.6)	138 (0.6)	202 (0.8)	131 (0.4)	139 (0.6)	126 (0.5)	331 (1.4)	24 (0.5)
Multiples, N (%)	1,804 (1.1)	435 (1.8)	235 (1.1)	361 (1.4)	194 (0.7)	155 (0.8)	156 (0.7)	210 (1.0)	58 (1.1)
Excluded due to missing variables, n (% of those with no other exclusions)	19,217 (11.7)	1,215 (5.2)	2,153 (10.0)	2,054 (8.3)	3,779 (13.2)	3,814 (19.6)	1,643 (7.7)	4,429 (21.8)	130 (2.6)
LBW status missing, N (%)	234 (0.1)	15 (0.1)	7 (0.0)	54 (0.2)	44 (0.2)	10 (0.1)	29 (0.1)	23 (0.1)	52 (1.0)
Missing any of the predictive model covariates, N (%)	19,117 (11.6)	1,208 (5.1)	2,153 (10.0)	2,028 (8.2)	3,779 (13.2)	3,812 (19.6)	1,626 (7.7)	4,425 (21.8)	86 (1.7)
Analysis population, N (% of delivered)	145,206 (81.9)	22,314 (90.5)	19,450 (87.9)	22,607 (88.7)	24,872 (84.1)	15,613 (65.1)	19,621 (84.5)	15,864 (68.9)	4,865 (92.0)
Fresh stillbirth, N (%)	2,268 (1.6)	416 (1.9)	205 (1.1)	340 (1.5)	354 (1.4)	220 (1.4)	225 (1.1)	449 (2.8)	59 (1.2)
LBW	1,282 (56.5)	195 (46.9)	98 (47.8)	131 (38.5)	216 (61.0)	180 (81.8)	174 (77.3)	252 (56.1)	36 (61.0)
Livebirth, N (%)	142,938 (98.4)	21,898 (98.1)	19,245 (98.9)	22,267 (98.5)	24,518 (98.6)	15,393 (98.6)	19,396 (98.9)	15,415 (97.2)	4,806 (98.8)
LBW	18,699 (13.1)	2,146 (9.8)	1,148 (6.0)	720 (3.2)	4,096 (16.7)	2,944 (19.1)	3,687 (19.0)	3,062 (19.9)	896 (18.6)
Total LBW, N (%)	19,981 (13.8)	2,341 (10.5)	1,246 (6.4)	851 (3.8)	4,312 (17.3)	3,124 (20.0)	3,861 (19.7)	3,314 (20.9)	932 (19.2)

*Abbreviations*: *DRC* Democratic Republic of the Congo, *MTP* medical termination of pregnancy, *LBW* low birth weight

^a^ Exclusions not mutually exclusive

We present the maternal and pregnancy characteristics in Table 2 for the analysis population. The majority of women included were 20–35 years old. Other maternal and pregnancy characteristics varied by site. Maternal and pregnancy characteristics by LBW status are provided in Supplement Table 1. Mothers of LBW infants were shorter (152 vs 155 cm), weighed less (49 vs 54 kg), and were less likely to have taken calcium or vitamin supplementation (14 vs 83%) than mothers who did not have a LBW infant. Mothers of LBW infants were also more likely to experience a complication of pregnancy, such as a hypertensive disorder (5.7 vs 1.7%), severe antepartum hemorrhage (2.3 vs 0.4%), severe infection of pregnancy (2.9 vs 1.1%), or fresh stillbirth (6.4 vs 0.8%).

**Table 2:** Maternal and pregnancy characteristics by site for the analysis population

Characteristics, n (%)	Overall *N* = 145,206	DRC *N* = 22,314	Zambia *N* = 19,450	Kenya *N* = 22,607	Guatemala *N* = 24,872	Belagavi *N* = 15,613	Nagpur *N* = 19,621	Pakistan *N* = 15,864	Bangladesh *N* = 4,865
Maternal age									
< 20	23,462 (16.2)	4,935 (22.1)	4,651 (23.9)	4,870 (21.5)	4,300 (17.3)	2,348 (15.0)	564 (2.9)	566 (3.6)	1,228 (25.2)
20–35	113,304 (78.0)	15,332 (68.7)	13,309 (68.4)	16,642 (73.6)	18,114 (72.8)	13,196 (84.5)	18,919 (96.4)	14,323 (90.3)	3,469 (71.3)
> 35	8,440 (5.8)	2,047 (9.2)	1,490 (7.7)	1,095 (4.8)	2,458 (9.9)	69 (0.4)	138 (0.7)	975 (6.1)	168 (3.5)
Maternal education									
No formal education	27,165 (18.7)	7,895 (35.4)	1,276 (6.6)	212 (0.9)	2,379 (9.6)	1,377 (8.8)	363 (1.9)	13,346 (84.1)	317 (6.5)
Primary/secondary	107,046 (73.7)	14,350 (64.3)	17,823 (91.6)	20,003 (88.5)	20,290 (81.6)	12,165 (77.9)	15,881 (80.9)	2,316 (14.6)	4,218 (86.7)
University +	10,995 (7.6)	69 (0.3)	351 (1.8)	2,392 (10.6)	2,203 (8.9)	2,071 (13.3)	3,377 (17.2)	202 (1.3)	330 (6.8)
Parity									
0	47,785 (32.9)	4,415 (19.8)	6,403 (32.9)	7,768 (34.4)	7,828 (31.5)	6,026 (38.6)	9,953 (50.7)	3,472 (21.9)	1,920 (39.5)
1	38,439 (26.5)	3,581 (16.0)	4,640 (23.9)	5,345 (23.6)	6,206 (25.0)	5,921 (37.9)	7,827 (39.9)	3,137 (19.8)	1,782 (36.6)
2	21,679 (14.9)	3,058 (13.7)	3,313 (17.0)	3,585 (15.9)	4,161 (16.7)	2,657 (17.0)	1,477 (7.5)	2,561 (16.1)	867 (17.8)
3	13,513 (9.3)	3,073 (13.8)	2,263 (11.6)	2,521 (11.2)	2,360 (9.5)	724 (4.6)	267 (1.4)	2,064 (13.0)	241 (5.0)
4 +	23,790 (16.4)	8,187 (36.7)	2,831 (14.6)	3,388 (15.0)	4,317 (17.4)	285 (1.8)	97 (0.5)	4,630 (29.2)	55 (1.1)
Maternal height in cm, mean (std)	154.2 (7.5)	156.5 (6.6)	158.0 (6.5)	160.3 (6.7)	146.8 (5.4)	152.0 (5.5)	152.7 (5.6)	154.5 (5.6)	151.2 (6.4)
Maternal weight in kg, mean (std)	53.7 (10.5)	52.6 (7.3)	59.5 (10.6)	61.1 (9.1)	56.5 (9.8)	46.8 (8.2)	46.1 (7.9)	50.1 (9.5)	52.8 (9.7)
Socioeconomic status score									
< 34	50,593 (34.8)	21,101 (94.6)	7,819 (40.2)	15,428 (68.2)	1,543 (6.2)	245 (1.6)	249 (1.3)	3,809 (24.0)	399 (8.2)
34–65	62,074 (42.7)	1,213 (5.4)	10,337 (53.1)	6,938 (30.7)	15,791 (63.5)	7,188 (46.0)	8,099 (41.3)	8,632 (54.4)	3,876 (79.7)
66 +	32,539 (22.4)	0 (0.0)	1,294 (6.7)	241 (1.1)	7,538 (30.3)	8,180 (52.4)	11,273 (57.5)	3,423 (21.6)	590 (12.1)
Previous livebirth									
Yes	93,797 (64.6)	17,231 (77.2)	12,578 (64.7)	14,211 (62.9)	16,329 (65.7)	9,246 (59.2)	9,352 (47.7)	12,054 (76.0)	2,796 (57.5)
No	3,624 (2.5)	668 (3.0)	469 (2.4)	628 (2.8)	715 (2.9)	341 (2.2)	316 (1.6)	338 (2.1)	149 (3.1)
No previous pregnancy lasting 20 + weeks	47,785 (32.9)	4,415 (19.8)	6,403 (32.9)	7,768 (34.4)	7,828 (31.5)	6,026 (38.6)	9,953 (50.7)	3,472 (21.9)	1,920 (39.5)
Number of antenatal care visits									
0	4,250 (2.9)	438 (2.0)	19 (0.1)	198 (0.9)	1,031 (4.1)	1 (0.0)	6 (0.0)	378 (2.4)	2,179 (44.8)
1–3	47,759 (32.9)	11,303 (50.7)	6,305 (32.4)	7,814 (34.6)	8,088 (32.5)	2,349 (15.0)	1,483 (7.6)	8,187 (51.6)	2,230 (45.8)
4 +	93,197 (64.2)	10,573 (47.4)	13,126 (67.5)	14,595 (64.6)	15,753 (63.3)	13,263 (84.9)	18,132 (92.4)	7,299 (46.0)	456 (9.4)
Iron supplement	138,318 (95.3)	21,165 (94.9)	19,405 (99.8)	22,369 (98.9)	23,818 (95.8)	15,546 (99.6)	19,559 (99.7)	12,572 (79.2)	3,884 (79.8)
Vitamin or calcium supplement	111,436 (76.7)	3,151 (14.1)	16,284 (83.7)	22,393 (99.1)	17,924 (72.1)	15,411 (98.7)	19,566 (99.7)	12,909 (81.4)	3,798 (78.1)
Hypertensive disorder	3,332 (2.3)	18 (0.1)	237 (1.2)	113 (0.5)	1,022 (4.1)	791 (5.1)	526 (2.7)	455 (2.9)	170 (3.5)
Severe antepartum hemorrhage	904 (0.6)	73 (0.3)	116 (0.6)	182 (0.8)	43 (0.2)	100 (0.6)	53 (0.3)	284 (1.8)	53 (1.1)
Severe infection during pregnancy	1,936 (1.3)	703 (3.2)	38 (0.2)	271 (1.2)	248 (1.0)	76 (0.5)	126 (0.6)	452 (2.8)	22 (0.5)

*Abbreviations*: *DRC* Democratic Republic of the Congo

The Pearson correlations for the variables included in the models were calculated (data not shown). Related variables include parity and previous livebirth (*r *=—0.72), SES and clinical site (*r* = 0.54), parity and age (*r* = 0.5), and previous livebirth and age (*r* =—0.45). The distribution of the outcome and predictors among the training dataset (*N* = 108,904) and test dataset (*N* = 36,302) were similar (data not shown).

**Fig. 2:**
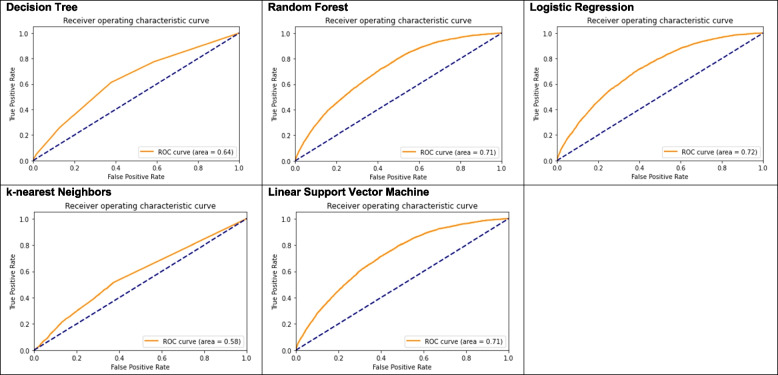
Receiver operator characteristic (ROC) curves for the predictive models

The predictive accuracy measures for the five models are provided in Table 3. The logistic regression model performed slightly better than the other models with an AUC score of 0.72 and an accuracy score of 61%. The positive predictive value (model precision) for logistic regression was 22% and the sensitivity (model recall) was 72%. The harmonic mean of precision and recall (model f1-score or model sensitivity) was 34%. For logistic regression, the default cut point value of 0.5 yielded the AUC and accuracy scores that were as good as the other cut point values (data not shown). The support vector machine linear kernel model performed similarly to the logistic regression and tree-based models. The polynomial and radial basis function support vector machines performed similarly to the linear support vector machine (data not shown). The k-nearest neighbors model results were different with an AUC value of 0.58 and an accuracy score of 83%. Although the accuracy for this model was higher, the positive predictive value and sensitivity for this model were 20% and 7%, respectively. The Receiver Operator Characteristic (ROC) curves for the predictive models are provided in Fig. 2.

**Table 3:** Predictive accuracy measures by predictive model

**Measures** ^a^	**Predictive model**				
	**Decision tree**	**Random forest**	**Logistic regression**	**K-nearest neighbors**	**Linear support vector machine**
Area under the curve score	0.64	0.71	0.72	0.58	0.71
Accuracy score	0.62	0.58	0.61	0.83	0.59
Among LBW babies					
Precision	0.21	0.21	0.22	0.20	0.21
Recall	0.61	0.74	0.72	0.07	0.75
f1-score	0.31	0.33	0.34	0.10	0.33
Permutation feature importance ^b^ – top variables in order of importance	1. maternal weight 2. hypertensive disorders	1. maternal weight 2. clinical site 3. hypertensive disorder 4. antenatal care 5. maternal height 6. antepartum hemorrhage 7. previous livebirth 8. parity	1. clinical site 2. maternal weight 3. antenatal care 4. hypertensive disorder 5. antepartum hemorrhage 6. severe infection during delivery 7. maternal height	1. maternal weight 2. maternal height 3. socio-economic status 4. antenatal care 5. parity 6. previous livebirth 7. maternal education 8. vitamin / calcium supplementation	1. clinical site 2. maternal weight 3. antenatal care 4. hypertensive disorder 5. antepartum hemorrhage 6. maternal education 7. severe infection during delivery
Hyperparameter values ^c^	Balanced class weights, Gini impurity criterion, minimum samples in a leaf = 2, minimum samples required for a split = 4, all others set to default	Balanced class weights, Gini impurity criterion, number of tree = 500, maximum depth = 8, all other set to default	Balanced class weights, L2 ridge regulation penalty, and maximum number of iterations for the solver to converge = 5000	Weights set by distance, leaf size = 20, all other set to default	Linear kernel, balanced class weights, regulation parameter (C) = 100, all other set to default

The binary outcome for the predictive models was LBW. Predictors included maternal age, maternal education, parity, maternal height, maternal weight, socioeconomic status (SES) score, previous livebirth, number of antenatal care visits, use of iron supplementation, use of vitamin or calcium supplementation, hypertensive disorders, severe antepartum hemorrhage, and severe infection during pregnancy. To develop the models, data were split into a training dataset (75% of available data) and test dataset (25% of available data). Hyperparameters were tuned using tenfold grid-search cross validation with scoring = ’roc_auc’. In addition to hyperparameter tuning, we varied the cut point for the probability used to classify an outcome as LBW for the logistic regression model from 0.1 to 0.9. We trained the models on the training data and validated the models on the test data

^a^ Predictive accuracy measures, including area under the curve (AUC) and receiver operator characteristic (ROC) curves, were produced using the test dataset. Precision, recall, and f1 scores were calculated using the classification_report method. Accuracy is (true positives + true negatives) / total. Precision is true positives / (true positives + false positives) and is also known as the Positive Predictive Value. Recall is the sensitivity or true positives / (true positives + false negatives). The f1-score is 2*(precision*recall) / (precision + recall); it is the harmonic mean of precision and recall

^b^ The permutation-based importance was implemented in Scikit-Learn as permutation_importance method. This method randomly shuffles each feature and computes the change in the model’s performance. The features which impact performance the most are the most important ones

^c^ For models which were tuned, the hyperparameter values presented are those in the tuned models

**Fig. 3:**
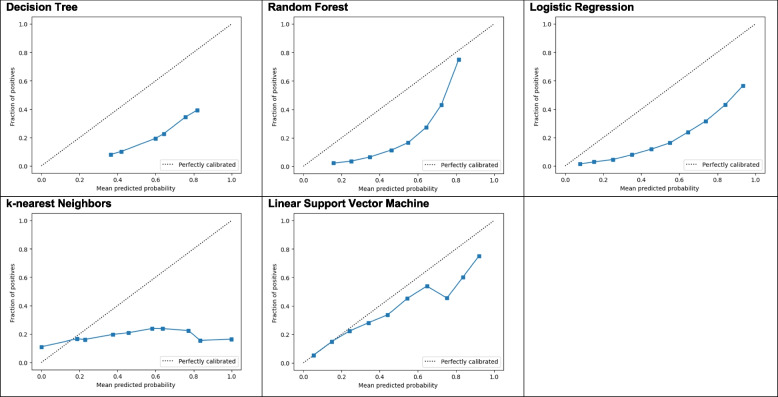
Calibration curves for the predictive models. The Y-axis is the true fraction of newborns who are low birth weight (LBW) and the X-axis is the model-predicted probability of being LBW. The worst performing model was k-nearest neighbors; the near-horizontal line for this curve indicates the model will predict a consistent LBW percentage of around 15% regardless of the true incidence of LBW. The best performing model was the linear support vector machine, which predicts nearly perfectly for the lowest incidence rates and begins to diverge around 40% incidence

Figure 3 depicts calibration curves for each of the predictive models. The Y-axis is the true fraction of newborns who are low birth weight (LBW) and the X-axis is the model-predicted probability of being LBW. The worst performing model was k-nearest neighbors; the near-horizontal line for this curve indicates the model will predict a consistent LBW percentage of around 15% regardless of the true incidence of LBW. The best performing model was the linear support vector machine, which predicts nearly perfectly for the lowest incidence rates and begins to diverge around 40% incidence.

**Fig. 4:**
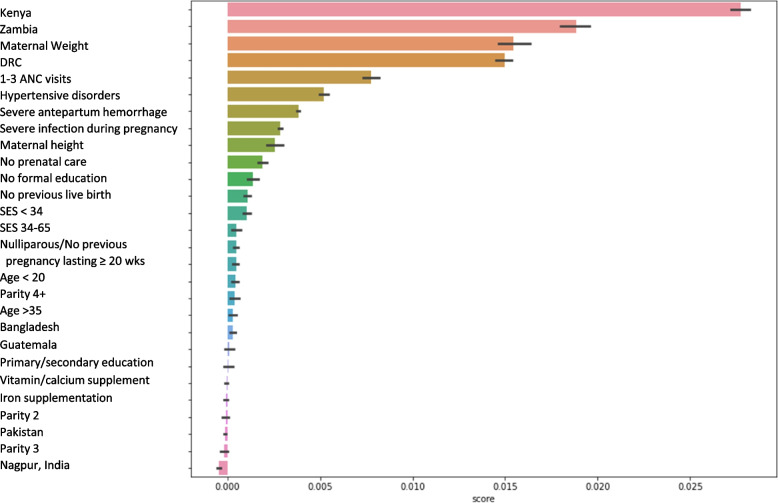
Permutation-based feature importance for the logistic regression model. The permutation-based importance was implemented in Scikit-Learn as permutation_importance method. This method randomly shuffles each feature and computes the change in the model’s performance. The features which impact the performance the most are the most important ones. The score is how the variable compares to other variables in the model. Thus, a high score for any level of a categorical variable indicates the entire variable is important. For clinical sites, the reference group is Belagavi, India. For maternal age, the reference group is 20–35 years. For maternal education, the reference group is University + . For parity, the reference group is parity of 1. For socio-economic status, the reference group is 66 + . For previous livebirth, yes is the reference group. For antenatal care visits, the reference group is 4 + visits

Figure 4 illustrates the permutation-based feature importance for the logistic regression model and partial dependency plots provide the directionality of these risk factors. For the logistic model, the most important variable relative to the other variables in predicting LBW was clinical site. The partial dependence plots show a higher probability of LBW for those not in the African sites, which coincides with the descriptive statistics that that the African sites had LBW rates a third of that of the Asian sites. Following clinical site, variables in order of importance that result in higher probability of LBW were lower maternal weight, 0–3 antenatal care visits, hypertensive disorder, severe antepartum hemorrhage, severe infection during delivery, and lower maternal height. The random forest and linear support vector machine models also found similar variables to be the most important in predicting LBW. The most important variables for each model are provided in Table 3. Table 4 provides regression coefficients and the model intercept for the logistic regression model.

**Table 4:** Logistic regression intercept and model coefficients for predictive model of low birth weight

Characteristics, n (%)	β
Intercept	13.20554481
Site	
Democratic Republic of the Congo	-0.67484249
Zambia	-0.92682525
Kenya	-1.40723953
Guatemala	-0.12309222
Belagavi, India	*ref*
Nagpur, India	0.0235633
Pakistan	0.014503
Bangladesh	-0.44466011
Maternal age	
< 20	0.09673194
20–35	*ref*
> 35	0.22481042
Maternal education	
No formal education	0.22003102
Primary/secondary	0.13738521
University +	*ref*
Parity	
0	0.15987423
1	*ref*
2	-0.12135872
3	-0.14800543
4 +	-0.26721066
Maternal height in cm	-0.02437492
Maternal weight in kg	-0.02930749
Socioeconomic status score	
< 34	0.0989827
34–65	0.07650165
66 +	*ref*
Previous livebirth	
Yes	*ref*
No	0.53308781
No previous pregnancy lasting 20 + weeks	0.15987423
Number of antenatal care visits	
0	0.68585889
1–3	0.51101636
4 +	*ref*
Iron supplement	0.14676482
Vitamin or calcium supplement	0.01635523
Hypertensive disorder	-1.18879701
Severe antepartum hemorrhage	-2.00835591
Severe infection during pregnancy	-1.07404103

## Discussion

We report a rate of LBW of 13.8% among the eight GN sites from 2017–2020, with a range of 3.8% (Kenya) and approximately 20% (in each Asian site). We found that mothers of LBW infants were more likely to experience a complication of pregnancy, such as hypertensive disorder, severe antepartum hemorrhage, severe infection of pregnancy, or fresh stillbirth. We used five predictive modeling strategies to identify pregnancy characteristics that predict the outcome of LBW infants. Of the five models tested, the logistic regression model performed the best with an AUC of 0.72 and an accuracy of 61%. All of the top performing models identified clinical site, maternal weight, antenatal care, hypertensive disorders, and severe antepartum hemorrhage as key variables in predicting LBW.

Our logistic regression model had reasonable performance to predict LBW using maternal and pregnancy characteristics prior to delivery. If we created a model that had predicted every outcome to be non-LBW, our accuracy rate would be 86%, given the 14% incidence of LBW in our sample; however, the recall of such a model would be 0. The recall, or sensitivity (proportion of true positives correctly identified), of our logistic regression model was 0.72. Since this model is intended to identify women with high-risk pregnancies for referral, a preferable model is one that errs on the side of over-identification (more false positives, lower specificity) than under-identification (more false negatives, or lower sensitivity). Our logistic regression model also had a precision, or positive predictive value (proportion of positives reported that are true positives) of 0.22. Lower performance for precision would increase the number of false positives, incorrectly identifying women as high-risk for LBW when they deliver a non-LBW infant. While over-predicting women who are at high-risk for delivering a LBW infant could put strain on an under-resourced health system, this is a reasonable allowance for a screening test to direct women to increased surveillance.

A comparison of our results to those from prior studies illustrates the importance of studying critical variables in various populations/datasets and comparing across models. Our logistic regression model performed similarly to the predictive model for LBW that included different factors associated with LBW from a case–control study in North India with 500 neonates. That study identified inadequate maternal weight gain, inadequate maternal protein intake, prior preterm infant, prior LBW infant, anemia and passive smoking as factors significantly associated with LBW [26]. Their predictive model had a sensitivity of 72% and specificity of 64%. Another model, using the Bangladeshi Demographic and Health Survey data identified alive child, education, height, region, twin child and wealth index as significant risk factors for LBW [27]. This logistic regression-based classifier had an AUC of 0.59 and accuracy of 87.6%. A United Arab Emirates study from a dataset of 821 women evaluated 30 machine learning algorithms for LBW classification, and found that logistic regression with SMOTE oversampling techniques achieved an accuracy of 90.24% and recall of 90.2%, with critical variables of diabetes, hypertension, and gestational age [8]. Developing the best predictive model may require expanding data collection to include additional relevant predictors from a variety of prospective modeling studies, which would lead to better overall model performance.

In low-resource settings where prenatal ultrasound is infrequently available to evaluate fetal weight, identification of LBW in advance of delivery using predictive modeling could have a substantial impact on care. Our top performing models identified a consistent cluster of variables available prior to delivery as important predictors of delivering a LBW infant, including low maternal weight, hypertensive disorder and severe antepartum hemorrhage. Maternal weight, hypertensive disorder, and severe antepartum hemorrhage are detectable at a time when referral is still feasible, and thus could be feasibly incorporated into a clinical tool to predict LBW. In particular, maternal malnutrition is a major, potentially modifiable predictor of LBW identifiable early in pregnancy. Limited antenatal care was also identified as a risk factor, but this variable is confounded by the higher number of preterm infants that are LBW, since preterm delivery truncates the usual number of antenatal care visits. We suspect that improving data collection in these key domains could improve the reliability of the predictive model. For example, inclusion of additional clinical information such as specific maternal blood pressure, might improve the accuracy of the predictive tools and thereby enhance the clinical utility of the predictive models. Our predictive modeling study is the first step in the development of a clinical tool to support decisions regarding referral of pregnancies at high risk for LBW in low-resource settings. While our study did not identify novel predictors of LBW, a clinical decision support tool incorporating these results could enhance care by standardizing referral decisions related to the anticipated delivery of a LBW infant.

Our study also identified clinical site as a consistent predictor of LBW across our top performing models. It is important to recognize that sites are not necessarily reflective of care across a country; future studies could consider analysis by geographic clusters with similar LBW rates as an alternative approach. Ultimately, the influence of site on prediction of LBW suggests that clinical tools to predict LBW should be developed within the site that they will be used. Our analysis provides a rubric for the development of similar tools in new sites, identifying an important set of predictors for collection that are not related to site, and indicating that a traditional logistic model is sufficient for analysis. Given the importance of site in the model, additional research could also focus on understanding how site differences are related to measurable characteristics, with replication of modeling with these new characteristics to improve predictive performance and reduce the importance of site identifiers in the model.

Our study has several notable strengths. We used high quality and robust prospectively-collected research data from the NICHD GN MNHR. This unique, population-based dataset contains maternal characteristics, pregnancy characteristics and delivery outcomes collected for a large number of women in seven different LMICs in Latin America, Africa and Asia. Due to the paucity of detailed health records in LMICs, the MNHR is an exceptional resource by which to build a predictive model. We assessed and compared the performance of five different predictive models using independent training and test data. The side-by-side comparison showed that the logistic regression model performed similarly to the random forest and linear support vector machine models, which is encouraging since logistic regression models are widely used and less complex. However, our study also had some limitations. We were limited by the data collected in the MNHR to build the predictive models. We had missing data for socio-economic status and maternal height (primarily Kenya) in early 2017. Maternal weight and clinical site were both predictors of LBW but were related with lower average weights in the Asian sites compared to higher average weights in Guatemala and the African sites. While using BMI instead of weight might account for some of the difference in weight across sites, we chose to maintain maternal height and weight as separate variables in our modeling since the MNHR includes sites where stunting or underweight are serious issues. We did not have information from clinical records such as maternal blood pressure, fundal height, or other features that might have improved the precision of our model. Despite these limitations, we believe that the variables collected approximate the typical data that might be readily available in a low-resource area, where clinical variables might be difficult to obtain. We limited the analysis to five different model types; other models such as extreme gradient boosting may have performed better.

## Conclusion

We identified several predictive modeling strategies that risk-stratify women in LMICs based on their risk of delivering a LBW infant using clinical variables readily available prior to delivery in low-resource settings. Our creation of these predictive models is an important first step in the development of a clinical decision support tool to prompt early referral of women at high-risk of delivering a LBW infant in LMICs. Such a clinical tool could facilitate standard referral of these women before delivery, directing the limited resource of advanced neonatal care to infants at highest risk. Timely, advanced care for LBW infants could reduce mortality and serious morbidity of these infants.

### Supplementary Information

**Additional file 1: Supplement Table 1.** Maternal and pregnancy characteristics by LBW status for the analysis population.

## Data Availability

The dataset analyzed for this study is available at the National Institute of Child Health and Human Development Data and Specimen Hub (https://doi.org/10.57982/t880-rf36).

## Abbreviation

LBW: Low birth weight
NICHD: National Institute of Child Health and Human Development
SGA: Small for gestational age
LMICs: Low- and middle-income countries
GN: *Eunice Kennedy Shriver* National Institute of Child Health and Human Development Global Network for Women’s and Children’s Health Research
MNHR: Maternal and Newborn Health Registry
MTP: Medical termination of pregnancy
SES: Socioeconomic status
CART: Classification and regression trees
AUC: Area under the curve
ROC: Receiver operator characteristic
