# Supplementary material for: Building a predictive model of low birth weight in low- and middle-income countries: a prospective cohort study
Source: BMC Pregnancy Childbirth. 2023 Aug 22;23:600. doi: 10.1186/s12884-023-05866-1 (PMC10464177; doi:10.1186/s12884-023-05866-1)
Supplement: Supplementary file 1 — Additional file 1: Supplement Table 1. Maternal and pregnancy characteristics by LBW status for the analysis population. [file 12884_2023_5866_MOESM1_ESM.docx]

**Supplement Table 1: Maternal and pregnancy characteristics by LBW status for the analysis population**

|  | **LBW** | |
| --- | --- | --- |
| **Characteristic, n (%)** | **Yes**  **N=19,981** | **No**  **N=125,225** |
| Maternal age |  |  |
| < 20 | 3,485 (17.4) | 19,977 (16.0) |
| 20-35 | 15,473 (77.4) | 97,831 (78.1) |
| > 35 | 1,023 (5.1) | 7,417 (5.9) |
| Maternal education |  |  |
| No formal education | 4,728 (23.7) | 22,437 (17.9) |
| Primary/secondary | 13,891 (69.5) | 93,155 (74.4) |
| University + | 1,362 (6.8) | 9,633 (7.7) |
| Parity |  |  |
| 0 | 8,186 (41.0) | 39,599 (31.6) |
| 1 | 5,298 (26.5) | 33,141 (26.5) |
| 2 | 2,518 (12.6) | 19,161 (15.3) |
| 3 | 1,437 (7.2) | 12,076 (9.6) |
| 4+ | 2,542 (12.7) | 21,248 (17.0) |
| Maternal height in cm, mean (std) | 151.5 (7.0) | 154.7 (7.4) |
| Maternal weight in kg, mean (std) | 49.4 (9.5) | 54.4 (10.5) |
| Socioeconomic status score |  |  |
| < 34 | 4,765 (23.8) | 45,828 (36.6) |
| 34-65 | 9,751 (48.8) | 52,323 (41.8) |
| 66+ | 5,465 (27.4) | 27,074 (21.6) |
| Previous livebirth |  |  |
| Yes | 11,140 (55.8) | 82,657 (66.0) |
| No | 655 (3.3) | 2,969 (2.4) |
| No previous pregnancy lasting  20+ weeks | 8,186 (41.0) | 39,599 (31.6) |
| Number of antenatal care visits |  |  |
| 0 | 898 (4.5) | 3,352 (2.7) |
| 1-3 | 7,489 (37.5) | 40,270 (32.2) |
| 4+ | 11,594 (58.0) | 81,603 (65.2) |
| Iron supplement | 21,165 (94.9) | 19,405 (99.8) |
| Vitamin or calcium supplement | 3,151 (14.1) | 16,284 (83.7) |
| Hypertensive disorder | 1,145 (5.7) | 2,187 (1.7) |
| Severe antepartum hemorrhage | 461 (2.3) | 443 (0.4) |
| Severe infection during pregnancy | 572 (2.9) | 1,364 (1.1) |
| Birth outcome |  |  |
| Fresh stillbirth | 1,282 (6.4) | 986 (0.8) |
| Livebirth | 18,699 (93.6) | 124,239 (99.2) |
